# Design and biofabrication of bacterial living materials with robust and multiplexed biosensing capabilities

**DOI:** 10.1016/j.mtbio.2022.100526

**Published:** 2022-12-24

**Authors:** Francesca Usai, Giada Loi, Franca Scocozza, Massimo Bellato, Ignazio Castagliuolo, Michele Conti, Lorenzo Pasotti

**Affiliations:** a Department of Electrical, Computer and Biomedical Engineering, University of Pavia, Via Ferrata 5, 27100, Pavia, Italy; b Department of Civil Engineering and Architecture, University of Pavia, Via Ferrata 3, 27100 Pavia, Italy; c Department of Information Engineering, University of Padua, Via Gradenigo 6b, 35131 Padua, Italy; d Department of Molecular Medicine, University of Padua, Via Gabelli 63, 35121 Padua, Italy

**Keywords:** Bioprinting, Engineered living materials, Biosensors, Synthetic biology, Engineered bacteria

## Abstract

The intertwined adoption of synthetic biology and 3D bioprinting has the potential to improve different application fields by fabricating engineered living materials (ELMs) with unnatural genetically-encoded sense & response capabilities. However, efforts are still needed to streamline the fabrication of sensing ELMs compatible with field use and improving their functional complexity. To investigate these two unmet needs, we adopted a workflow to reproducibly construct bacterial ELMs with synthetic biosensing circuits that provide red pigmentation as visible readout in response to different proof-of-concept chemical inducers. We first fabricated single-input/single-output ELMs and we demonstrated their robust performance in terms of longevity (cell viability and evolutionary stability >15 days, and long-term storage >1 month), sensing in harsh, non-sterile or nutrient-free conditions compatible with field use (soil, water, and clinical samples, including real samples from *Pseudomonas aeruginosa* infected patients). Then, we fabricated ELMs including multiple spatially-separated biosensor strains to engineer: level-bar materials detecting molecule concentration ranges, multi-input/multi-output devices with multiplexed sensing and information processing capabilities, and materials with cell-cell communication enabling on-demand pattern formation. Overall, we showed successful field use and multiplexed functioning of reproducibly fabricated ELMs, paving the way to a future automation of the prototyping process and boosting applications of such devices as in-situ monitoring tools or easy-to-use sensing kits.

## Graphical abstract

**Figure:**
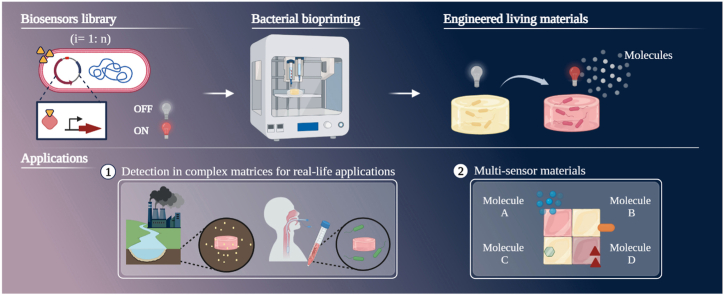

## 1 Introduction

The intertwined adoption of synthetic biology and bioprinting techniques has the potential of realizing engineered living materials (ELMs) with unprecedented complexity, made by the integration of abiotic components and living cells [1,2].

Complexity is enabled by the design of novel biological functions in engineered cells, such as microbes exhibiting sensing of environmental cues, actuation, and information processing tasks, and the precise spatial organization enabled by the fabrication of functional ELM structures with desired mechanical properties via additive manufacturing methods [[3], [4], [5]].

The fabrication of functional bacteria-laden structures can address several unmet needs of our society in diverse applications, such as therapeutics, environment, and industrial manufacturing [6,7], using rational engineering techniques to tune biological and mechanical properties of ELMs to meet different specifications [8,9].

Extrusion-based bacterial printing using custom-built or commercial bioprinters was demonstrated on wild-type [[10], [11], [12], [13], [14]] and engineered [[15], [16], [17], [18], [19], [20], [21]] bacterial species. Stereolithographic and inkjet-based bioprinting of engineered strains were also reported in recent studies, the latter being used to fabricate the sole abiotic component [22,23]. These works reported materials for different applications, such as food manufacturing, current generation, biofilm design and characterization, bioremediation, sensing of pathogens or pollutants, and on-demand bioproduction of polymers and drugs.

Biosensor design is a major task in synthetic biology that can rely on predictable programming of whole-cell sensing microorganisms. Synthetic circuits containing a transcriptional regulator activating or repressing its cognate promoter in a signal-dependent fashion constitute a genetic biosensor with a transcriptional output. Reporter genes are assembled downstream of the regulated promoter to provide color, fluorescence or light as readout [24]. The input-output characteristic of such biosensors is nonlinear with saturation, usually showing a Hill function shape, thus requiring tuning steps to match the desired variation range of the input [25,26]. Compared with traditional detection methods, advantages of genetically-encoded biosensors generally include low costs, high portability and usability, and minimal requirement of sample preprocessing, specific equipment, trained personnel, and output post-processing [27].

Key criteria, shared by many target applications, need to be satisfied to boost the field use of biosensors [28]: specificity, reproducible functioning, reasonable detection limit, ease of use in terms of assembly and readout detection, rapid response, biocontainment, and longevity also including evolutionary stability of the living component. In addition, quantitative rather than qualitative outputs and multiplexed detection by multiple sensing strains in the same device can benefit several applications [29].

These features have been investigated by biosensor circuit programming and material design to obtain field-deployable non-bioprinted sensor devices [[30], [31], [32]], e.g., long-term storage of bacteria-laden materials for sensing in environmental water samples that retained their functionality over time albeit with increasing output variability. A few studies on bioprinted ELM sensors also tested features relevant to field use, such as spore-laden materials that survived to stress conditions and in which germination can be triggered on-demand [19]. Multi-strain bioprinted ELMs were also reported in a few studies, in which stacked layers [15] and planar structures [18] of different strains or even distinct species were printed for compartmentalized operations such as bioproduction [33,34].

Although promising performance of field-deployable biosensors have been reported in recent studies, demonstrative work on field-use and multi-input/multi-output function design in bioprinted ELMs is still missing, and could enable automated fabrication processes for rapid prototyping and streamlined production of customized sensing devices. Expanding the functional and application repertoire of bioprinted ELM-based sensing devices is expected to bring significant advantages to the biosensor field, such as the realization of easy to use multi-functional materials for different applications, obtained via highly reproducible and parallelized biofabrication techniques. ELM devices are also expected to provide advantages over cell-free expression-based sensing methods, which include promising portable testing devices but are more sensitive to matrix effects, e.g., inhibitors, than whole-cell sensing systems, although recent efforts demonstrated that a mitigation of such effects could be achieved [35].

In this work, we use engineered bacteria capable of extracellular molecule (e.g., signaling molecule, antibiotic) sensing as model systems and an extrusion-based bioprinting platform to investigate two field-relevant unmet needs for biosensing ELM construction: functionality, reproducibility and tunability of ELMs, and sensing in harsh environmental or clinical samples. Fabrication of multi-strain materials is also used to construct ELMs capable of quantitative or semi-quantitative readout, multi-input multi-output detection and cell-to-cell communication in the same ELM. These analyses are currently unexplored in ELMs and will pave the way to a more rational application-oriented material engineering approach, as well as supporting design-build-test cycles to develop new functional living materials.

## 2 Methods

### 2.1 Reagents, media, and strain construction

#### 2.1.1 Bacterial strains

The engineered strains used in this work are reported in Table 1. The *Escherichia coli* strains are derivatives of TOP10, MG1655, and MG1655-Z1, and the *Bacillus subtilis* strain is a derivative of JH642. MG1655-Z1 is similar to MG1655, with additional constitutive overexpression cassettes for LacI and TetR, which make the P_LlacO1_ and P_LtetO1_ promoters inducible.

**Table 1:** Engineered strains used in this work.

Engineered strain	Chassis	Insert^a^	Vector^b^	Description	Source
HCred	TOP10	I13521	pSB1A2	P_LtetO1_-driven constitutive RFP (high-copy plasmid)	MIT Registry
MCred	TOP10	I13521	pSB3K3	P_LtetO1_-driven constitutive RFP (medium-copy plasmid)	[37]
MCgreen	TOP10	J107040	pSB3K3	P_LtetO1_-driven constitutive GFP	This work
TET-MCred	MG1655-Z1	I13521	pSB3K3	Tc/aTc-inducible RFP	[37]
TET-MCyellow	MG1655-Z1	J107263	pSB3K3	Tc/aTc-inducible xylE	This work
TET-MCvai	MG1655-Z1	K516210	pSB3K3	Tc/aTc-inducible luxI	[36]
LAC-MCred	MG1655-Z1	J107010	pSB3K3	IPTG-inducible RFP	[37]
SensRegRFP-MC	MG1655-Z1	J107053	pSB3K3	VAI-inducible RFP with luxR controlled by IPTG-inducible promoter	[36]
LUX_lac_-MCred	MG1655	J107053	pSB3K3	VAI-inducible RFP with luxR expressed by non-repressed P_LlacO1_	This work
LUX_100_-MCred	MG1655	J107264	pSB3K3	VAI-inducible RFP with luxR expressed by the J23100 promoter^c^	This work
LUX_110_-MCred	MG1655	J107265	pSB3K3	VAI-inducible RFP with luxR expressed by the J23110 promoter	This work
LUX_105_-MCred	MG1655	J107266	pSB3K3	VAI-inducible RFP with luxR expressed by the J23105 promoter	This work
LUX_116_-MCred	MG1655	J107267	pSB3K3	VAI-inducible RFP with luxR expressed by the J23116 promoter	This work
LUX_117_-MCred	MG1655	J107268	pSB3K3	VAI-inducible RFP with luxR expressed by the J23117 promoter	This work
OL1-SensRegRFP	MG1655-Z1	J107053 andK516210	pSB3K3 andpSB4C5	VAI-inducible RFP with luxR controlled by an IPTG-induciblepromoter (with additional plasmid causing cell load)	[36]
PB5741	JH642	-d	–	IPTG-inducible γ-PGA synthesis	[47]

a DNA sequences are available as entries in the MIT Registry of Standard Biological Parts (http://partsregistry.org) using the provided codes, in which the “BBa_” prefix is omitted.

b pSB1A2, pSB3K3, and pSB4C5 are high-, medium-, and low-copy vectors, respectively, and their sequences are available in the Registry of Standard Biological Parts.

c The J231xx promoter series is a library of constitutive promoters of different strengths, widely used in synthetic biology and referred to as the Anderson promoter collection, accessible in the Registry of Standard Biological Parts.

d Chromosomally integrated strain with no plasmids.

#### 2.1.2 Reagents

Ampicillin (100 ​μg/ml), kanamycin (25 ​μg/ml), chloramphenicol (12.5 ​μg/ml), and erythromycin (2 ​μg/ml) were used to select *E. coli* with genetic circuits in the pSB1A2, pSB3K3 and pSB4C5, and engineered *B. subtilis*, respectively. The *Vibrio fischerii* autoinducer N-3-oxohexanoyl-l-homoserine lactone (VAI, #K3007, Sigma Aldrich), *Pseudomonas aeruginosa* autoinducer (PAI, #O9139, Sigma Aldrich), Isopropyl-β-d-1-thiogalactopyranoside (IPTG, #I1284, Sigma Aldrich), anhydrotetracycline (aTc, #631310, Clontech), and tetracycline (Tc) were used as biosensor inputs. Sodium alginate (#W201502, Sigma Aldrich) and gelatin (#G9391, Sigma Aldrich) were used as hydrogel components. Catechol (#C9510, Sigma Aldrich) was used at 10 ​mM for XylE yellow staining.

#### 2.1.3 Cloning

Plasmid construction was performed with the BioBrick Standard Assembly procedure, starting from publicly available plasmids from the 2008–2011 distributions of the Registry of Standard Biological Parts. Briefly, plasmid DNA previously purified via Plasmid kit (Macherey Nagel) was digested with *Eco*RI/*Xba*I/*Spe*I/*Pst*I restriction enzymes (Thermo Fisher). Digestion products were separated on 1% agarose gel electrophoresis, extracted via PCR Cleanup kit (Macherey Nagel), and assembled using T4 ligase (Thermo Fisher). Enzymes and kits were used according to manufacturer instructions. *E. coli* transformation was done by heat shock at 42 ​°C, using chemically competent cells. Strains were routinely grown at 37 ​°C, 220 ​rpm, in 5 ​ml of L-broth (LB) medium (1% NaCl, 1% tryptone, 0.5% yeast extract, 1.5% agar for solid media) and long-term stored at −80 ​°C in 20% glycerol stocks. Sequencing was performed by Eurofins Genomics (Ebersberg, Germany).

### 2.2 Bioprinting

#### 2.2.1 Hardware and software

The extrusion-based CELLINK INKREDIBLE+ ​bioprinter (Cellink AB, Sweden), equipped with two printheads (PHs), was adopted. Structures were designed as CAD files using the Autodesk Inventor Pro software (Autodesk, United States), then the CAD model was sliced using Slic3r, an open-source slicing software. During the slicing process, printing parameters (e.g., layer height, perimeter, printing speed, infill percentage) were defined. After slicing, the specific set of instructions (i.e., G-code) was created and constructs were 3D printed.

#### 2.2.2 Hydrogel

An 8% (w/v) sodium alginate and 4% (w/v) gelatin hydrogel was prepared one day before use by dissolving gelatin powder in pre-warmed PBS (70 ​°C) and then dissolving sodium alginate. The hydrogel was supplemented with antibiotics as required, and stored at 4 ​°C.

#### 2.2.3 Printing procedure

Unless otherwise indicated, bacteria from a saturated culture, grown in selective LB medium at 37 ​°C, 220 ​rpm, were centrifuged (4000 ​rpm, 10 ​min); the supernatant was removed and the pellet was resuspended with the same volume of 2-fold concentrated LB medium. The resuspended culture was mixed at 1:10 ratio with the hydrogel at room temperature using two syringes with a Luer connector. The prepared hydrogel-bacteria mixture, referred to as *bioink*, was loaded into the printer cartridge(s) and the printing was performed on Petri dishes using a 0.41-mm nozzle, pressure from 15 to 25 ​kPa, and an extrusion speed of 800 ​mm/min. After printing, the construct was crosslinked for 5 ​min using 2% (w/v) calcium chloride, which was removed with a pipette and the structure was moved onto selective LB agar. The structure was then moved to fresh LB agar at specific time points to extend cell viability and crosslinking was occasionally refreshed to maintain structural integrity of printed construct.

#### 2.2.4 Tested shapes

Table S1 summarizes the different structure geometries printed for this study.

### 2.3 ELM characterization

#### 2.3.1 Microscopy

Fluorescent protein expression by printed bacteria was observed with the Eclipse 80i microscope (Nikon). Fluorescence images were taken via the Image-Pro Plus software. The TRITC (excitation/emission: 520.5/582 ​nm) and FITC (495/528 ​nm) channel setups were used to measure the red and green fluorescence of mRFP1 and GFPmut3b, respectively. ELM slices were cut with a scalpel and they were imaged on a microscope slide with a 4× objective.

#### 2.3.2 Cell viability

Bacterial cell viability was quantified via colony counts in dissolved ELMs. A pre-weighted structure was immersed in 2 ​ml of 0.1 ​M sodium citrate in a 15-ml tube and incubated at room temperature for 1 ​h on a rolling shaker set at slow rotation. Serial dilutions were plated on selective LB agar and incubated at 37 ​°C overnight. Colony count was multiplied by dilution factor and expressed as colony forming units (CFUs) per mg of structure. No detectable toxicity by sodium citrate, in terms of CFUs, was observed in preliminary tests in which liquid cultures were incubated as above in 0.1 ​M sodium citrate or PBS, used as control, before plating (data not shown).

#### 2.3.3 Preliminary biosensor tests in liquid cultures

Fluorescence assays were carried out in a microplate reader (Infinite F200Pro, Tecan), measuring growth (OD; absorbance at 600 ​nm) and red fluorescence (RFP; excitation/emission: 535/620 ​nm) every 5 ​min [36]. Strains were grown overnight (37 ​°C, 220 ​rpm) in 0.5 ​ml of selective M9 supplemented medium (M9 salts - #M6030, Sigma Aldrich - 11.28 ​g/l, thiamine hydrochloride 1 ​mM, MgSO_4_ 2 ​mM, CaCl_2_ 0.1 ​mM, casamino acids 0.2%, glycerol 0.4%). They were 100-fold diluted in 200 ​μl of the same medium and incubated in 96-well plates at 37 ​°C with linear shaking (3 ​mm, 5 ​s before sampling). Inducers (VAI, PAI, aTc or Tc) were added in the 200 ​μl cultures at the specified concentrations. Data analysis included background subtraction from raw absorbance and fluorescence by using media and a non-fluorescent culture, respectively; biosensor output was computed as RFP/OD, expressed in arbitrary units (AU) of per-cell fluorescence. Quantification of unknown PAI concentration in *P. aeruginosa* contaminated samples was carried out using a similar protocol with the LUX_lac_-MCred strain, including a standard PAI calibration curve as previously described [37]. After considering the dilution of unknown samples in the biosensor cultures, the lower detection limit was about 200 ​nM of PAI.

#### 2.3.4 Test of biosensor ELMs

Unless differently indicated, biosensing assays with ELMs were carried out by placing patch structures (20 ​× ​20 ​× ​1.4 ​mm, see Table S1), at specific time points, on selective LB agar containing chemical inducers to guarantee full induction of the respective expression systems (VAI and PAI at 400 ​nM, aTc and Tc at 200 ​ng/ml, IPTG at 100 ​μM), and incubated at 37 ​°C. RFP output was observed by visual inspection after a specified time. Overnight refers to 16–24 ​h. XylE assays were performed analogously, with the exception that the structures had letter geometries (Table S1) instead of patches, were removed from the plate and catechol was added to start the enzymatic reaction yielding the yellow product 2-hydroxymuconic semialdehyde. Induction was carried out at different times to test the robustness of sensing ELMs and day 0 refers to the day of bioprinting. When indicated, structures printed at day 0 were stored at 2–8 ​°C on LB agar plate for the specified time until further use.

#### 2.3.5 Biosensing tests in environmentally relevant samples

VAI detection by sensing ELM (LUX_lac_-MCred sensor strain) was tested in commercial topsoil (Esselunga, Italy) and tap water. ELMs were printed and incubated in LB agar as above. At day 1, VAI was added to the soil by adding about 10% (w/w) water containing the autoinducer at a 400 ​nM concentration, and structures were placed in the induced soil. The same concentration of VAI was added to 100 ​ml of tap water and structures were immersed overnight. Incubation was carried out at room temperature.

#### 2.3.6 Biosensing tests in clinically relevant samples

The VAI-sensing ELM was also used to detect PAI in supernatants of *P. aeruginosa* isolates or bronchial aspirates. Clinical isolates of *P. aeruginosa*, collected from patients affected by cystic fibrosis (Padua University Hospital), were cultured in LB medium for 24 ​h (37 ​°C, 220 ​rpm), cells were spun down and supernatants were 0.2-μm filtered. Bronchial aspirate samples from patients (Padua University Hospital) affected by Gram positive or Gram negative bacterial infections (including *P. aeruginosa*) were diluted at a 1:1 ratio with sputasol (Oxoid), centrifuged and the supernatants collected. Supernatants of clinical isolates or bronchial aspirate samples were stored at −20 ​°C until further use. Biosensing experiments were carried out by pipetting 200 ​μl of samples on ELMs with a cut section of LB agar providing additional nutrients (see Results section for details) and incubating these structures at 37 ​°C overnight. Bronchial aspirate samples with no *P. aeruginosa* were tested with and without spiked PAI at 400 ​nM.

When indicated, structures printed at day 0 were stored at 2–8 ​°C on LB agar plate for the specified time until further use.

### 2.4 Image analysis

#### 2.4.1 Shape fidelity of printed ELMs

Images of 20 ​× ​20 mm grid structures with different height and distance between filaments (see Table S1) were analyzed using ImageJ [38] to assess the fidelity of strand size and distance between the filaments in terms of coefficient of variation (CV) in at least 30 values measured from the same structure and different structures. The fidelity of square pores in grids was quantified by computing $\mathrm{Pr}=\frac{L^{2}}{16∙A}$, where *L* and *A* indicate pore perimeter and area, respectively [39]. The Pr index is 1 for square pores, lower than 1 for circular pores (0.785 for perfect circles), and higher than 1 for irregularly gelated structures.

#### 2.4.2 Separation of strains in multi-bioink ELMs

Analysis of fluorescence microscopy pictures with ImageJ was used to measure separation and cross-contamination between adjacent sections of the same material, composed of different strains expressing red and green fluorescent proteins.

#### 2.4.3 Quantification of red pigmentation

Images of biosensing patches were cropped and the intensity of red pigmentation was computed using the CIELAB system, a color space based on three opponent color channels, namely black/white (L∗), red/green (a∗), and yellow/blue (b∗). The a∗ values were quantified using a custom Python script (functions from the *skimage* and *cv2* modules) and were used to compare the intensity level of red in the red/green channel coordinates among different images. The a∗ values shown in graphs are relative to pictures acquired under similar illumination conditions, except for the experiments with bronchial aspirates that were carried out in a different institute and a different acquisition setup was adopted.

Data of red pigmentation intensity are reported with the specified number of replicates carried out using different bacterial culture batches prepared in the same day and in different days, as indicated. Batch-to-batch and day-to-day variability in the VAI-sensing ELM response were quantified in terms of average CV across all the VAI concentration conditions.

When indicated, red color data derived from pictures were confirmed by red fluorescence measurements of ELMs that were cut and transferred into individual wells of 96-well plates. Red fluorescence was acquired as described in section 2.3.3, using 3-by-3 square-filled acquisitions per well (RFP gain ​= ​30) and averaging the obtained values.

#### 2.4.4 Statistical analysis

Unpaired *t*-test was used to evaluate statistical differences between two samples using Microsoft Excel. Two-way ANOVA with interactions was adopted to evaluate the impact of two variables (namely, temperature and presence of agar medium) in the same experimental study, using the Matlab R2017b (MathWorks, Natick, US) *anovan* function. Pearson correlation coefficient (r) and its relative p-value (p) were computed with the Matlab *corrcoef* function. A p-value cutoff of 0.05 was adopted to evaluate statistical significance.

## 3 Results and discussion

### 3.1 Bioprinting workflow

#### 3.1.1 Workflow definition

**Fig. 1:**
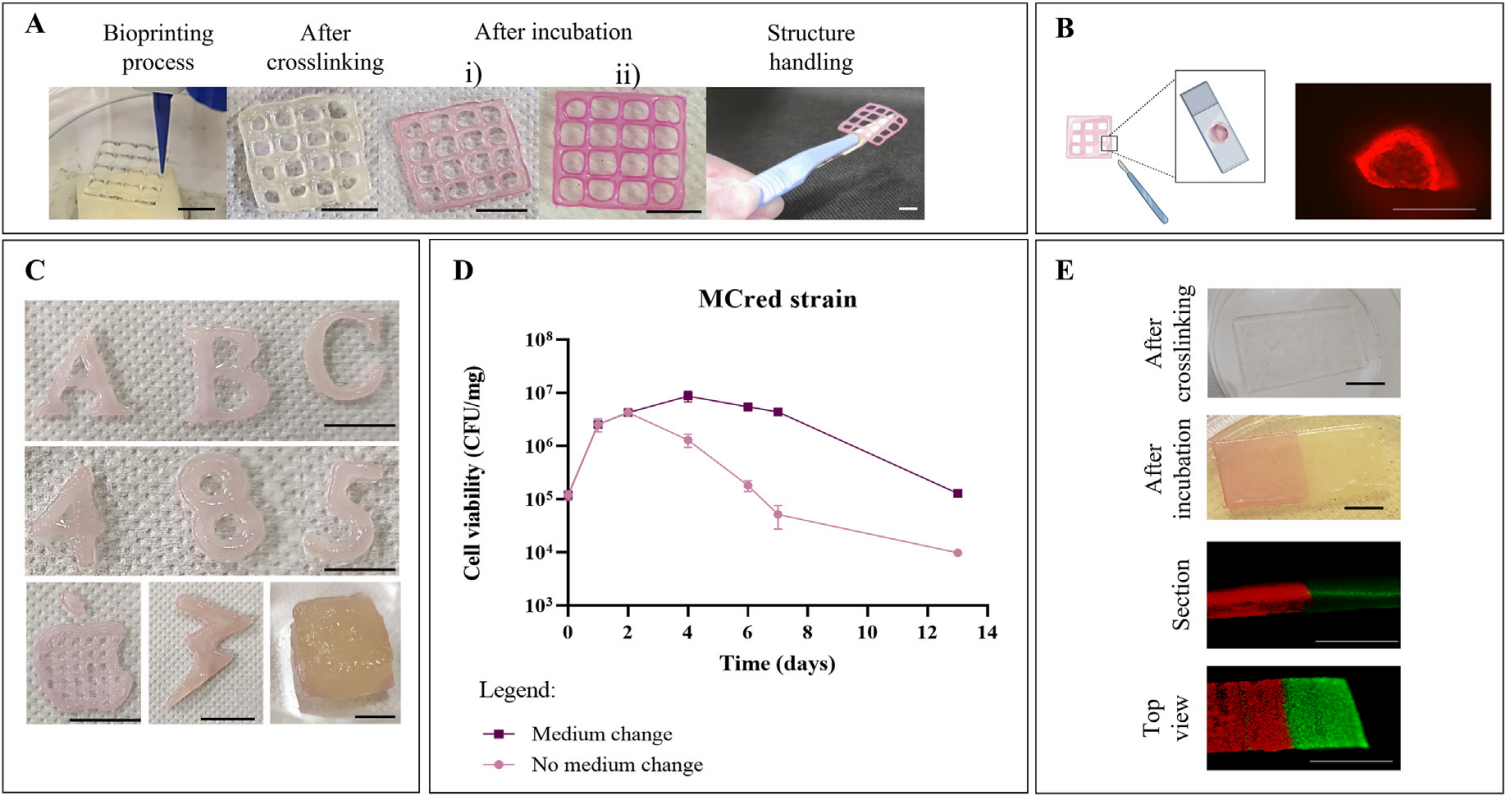
Bacteria-laden ELMs with different shapes showing cell viability and protein expression. A) ELMs pictures during bioink extrusion, just after printing and crosslinking (day 0), after overnight incubation (day 1), and handled after removal from LB agar (day 1). The MCred (i) and HCred (ii) strains were the living components in this panel. B) Microscopic image of the section of a strand in a 20 ​× ​20 ​× ​1 mm grid structure with MCred strain. The microscopy picture was acquired using the TRITC filter channel, 150 ​ms exposure. C) Complex structures including engineered *E. coli* (MCred) and visualized at day 1. D) Cell viability profile in terms of CFUs per mg of ELM, using MCred printed in a set of grid structures (all printed on the same day) which were weighted, entirely dissolved, diluted as appropriate and plated to enable colony count. Solid line represents the mean of three replicates of the same bioprinting batch and error bars represent the standard deviation. The two curves correspond to ELMs that were incubated on the same solid medium for the specified time or subcultured by moving the structure on fresh medium at day 2 and day 5. E) Two-strain ELM (40 ​× ​20 ​× ​1.2 ​mm) including the MCrfp (left square) and MCgfp (right square) strains. The ELM is shown just after bioprinting and crosslinking, and after overnight incubation, in which pigmentation by RFP and GFP can be observed at naked eye. Microscopy images of this ELM are shown below as section and top view, used to assess separation and cross-contamination between the two bioinks. Scale bar: 10 ​mm for panels A, C, E; 1 ​mm for panel B.

A commercial extrusion-based bioprinting platform was used to define a simple bacterial bioprinting procedure achieving microbe-laden structures with predictable shapes, sufficiently tough to be easily handled downstream of the printing steps (Fig. 1A). Several parameters and conditions were screened during preliminary tests to optimize the process, also inspired by previous experience on bacteria or other organisms [15,40,41], as described in the Supplementary Material (Text S1 and Fig. S1). Bacteria bearing a constitutive RFP expression system in high- or medium-copy plasmid (HCred and MCred strains) were used in these tests to guarantee the observation of red color development at naked eye upon successful bacterial growth and protein expression (Fig. 1A).

The workflow could yield structures of different shapes and scales, described in Table S1. MCred bacteria-laden structures were successfully printed, obtaining circular, square, sharp, and hollow features (Fig. 1A–C), with 10- to 50-mm length and structure height from <1 ​mm up to 5.1 ​mm (17 layers). All of them showed red pigmentation confirming RFP expression, with the strain bearing the expression cassette in high-copy producing a more intense red color than the medium-copy one, as expected (Fig. 1A).

Shape fidelity and reproducibility of printed structures were also quantified and demonstrated that the bioprinting process yields structures with a reasonable predictability at the sub-millimeter scale (Text S2 and Fig. S2).

#### 3.1.2 Bacterial growth and protein expression in ELMs

The spatial distribution of protein expression in ELMs was investigated by analyzing sections of bioprinted structures with fluorescence microscopy. As previously observed in other setups [19], fluorescence is more intense at the edge of the structure (Fig. 1B), although bacteria are homogeneously mixed in the bioink. Intensity decreases sharply at a depth of about 200 ​μm. Since strong fluorescence was observed on top of the construct opposite to the LB agar, RFP heterogeneity is not caused by nutrient limitation preventing growth or gene expression. Rather, oxygen limitation is likely to occur at the center of the structure, reducing bacterial proliferation and RFP maturation [42].

Bioprinting of the HCred and MCred strains in grid structures (Table S1) and incubation on solid media supported a 100- to 1000-fold increase of bacterial density after one day and up to ∼10^7^ ​cells per mg (Fig. 1D and Fig. S1B). The CFU/mg curve increases up to day 2–3, after which cell viability decreases, probably due to the lack of nutrients or accumulation of toxic waste products in solid media. Cell growth was then optimized via a subculturing procedure, in which ELMs were moved onto fresh media at specific time points (day 2 and 5) and viability was significantly extended (Fig. 1D). A drop in cell viability was still observed, probably due to material overcrowding or accumulation of toxic products, but the cell density profile showed a much slower decline than in the no-subculturing condition, reaching a density comparable with the initial one after 2 weeks. This optimization of cell viability within ELMs is promising for the reliability of the target biological function over time during continuous functioning of the ELM. ELMs from the same bioprinting batch showed a low variability in the initial cell density in ELM and in the growth profiles, demonstrating that a high reproducibility persists in cell viability during the bioprinting process (Fig. 1D). Moreover, constitutive RFP-producing bacteria extracted from dissolved ELM formed visibly red colonies, demonstrating a high evolutionary stability over 2 weeks with no detectable mutants for MCred (data not shown).

#### 3.1.3 Printing ELMs with multiple strains

We finally tested the fabrication of ELMs including multiple bioinks by printing materials with adjacent strains on the same layers. After extrusion, crosslinking was carried out on all the deposited bioinks to generate a unique ELM. We printed two strains constitutively expressing different fluorescent proteins (RFP and GFP). Red and green fluorescence could be conveniently observed with the microscope to assess the absence of cross-contamination between the two compartments and the presence of a net separation between them (Fig. 1E). We quantified the cross-contamination by detecting green and red fluorescence in the compartments expressing RFP and GFP, respectively, on the ELM surface. Results showed that GFP cross-contamination in the RFP compartment was as low as 0.1%, while no red fluorescence was detected in the GFP compartment. A section of the two-strain ELM also showed net separation between adjacent compartments, demonstrating the reliability of multi-strain ELMs (Fig. 1E).

The simple fabrication workflow herein tested thus allows for reproducibility in the construction of ELMs with predictable shapes, cell viability and functioning, and many of its steps are also compatible with automation.

### 3.2 Single-strain biosensing materials

#### 3.2.1 Characterization of a VAI-sensing ELM

**Fig. 2:**
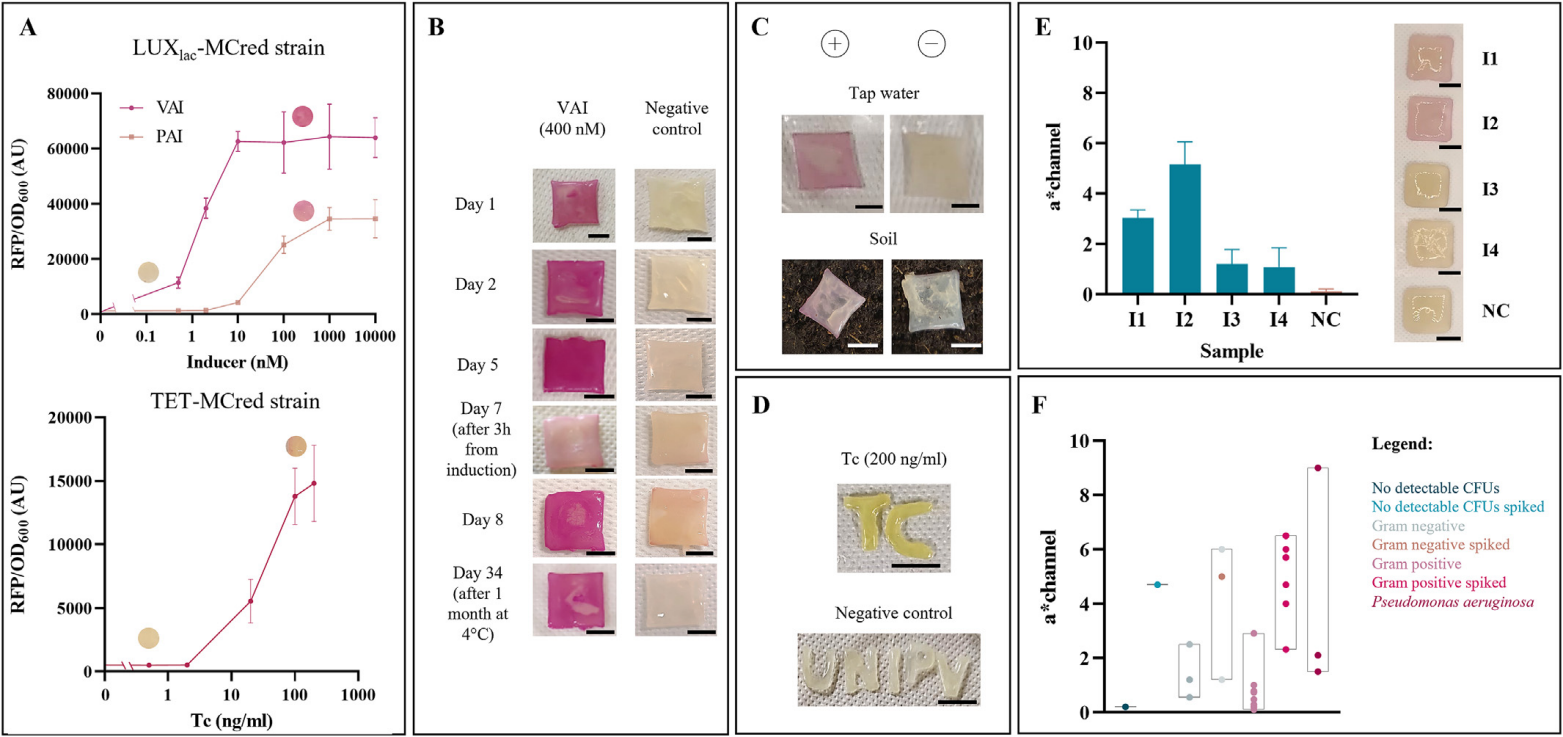
Single-strain ELMs for detection of signaling molecules and antibiotics. A) Input-output transfer functions at the steady-state, with per-cell RFP fluorescence as output, of the VAI-sensing strain (LUX_lac_-MCred) and the Tc-sensing strain (TET-MCred) in liquid cultures. Data points represent the average values of three independent measurements and error bars represent the standard deviations. The visualized pigmentation of bioprinted structures after overnight incubation on LB agar is also shown in the plots for the non-induced and fully induced conditions. B) Patches of the VAI-sensing ELM (LUX_lac_-MCred) after overnight incubation on VAI-containing LB with induction carried out at different days of continuous ELM culturing. Two patches are also shown after 3 ​h incubation from induction time instead of overnight, and upon overnight incubation after conservation in a regular fridge for 1 month. Overnight incubation was carried out at 37 ​°C and the induction was performed using 400 ​nM VAI. C) Patches of the VAI-sensing ELM (LUX_lac_-MCred) after overnight incubation in tap water and soil containing VAI. ELMs were incubated on LB agar overnight after printing, then they were removed from the solid media and applied to the tap water or soil, as indicated. Pictures were taken at day 1 (soil) and 2 (water) upon incubation at room temperature (see text). Tap water contained 400 ​nM VAI and soil contained ∼10% of water with 400 ​nM VAI. D) Tc-sensing ELM (TET-MCyellow) after overnight incubation at 37 ​°C on LB containing the indicated inducers, and then stained with catechol. Pictures were taken after overnight incubation and after 1 ​min from the addition of catechol. Scale bar: 10 ​mm. E) ELM (LUX_lac_-MCred) response upon PAI sensing from filter-sterilized supernatants of four *P. aeruginosa* clinical isolates (I1–I4). The a∗ values are reported to quantify the red pigmentation and the corresponding pictures are shown. Negative control (NC) refers to the supernatant of an MG1655-Z1 strain grown at 37 ​°C, 220 ​rpm overnight, not producing PAI. ELMs were stored at 2–8 ​°C for 1 day before use. Bars represent mean values and error bars represent standard deviations (N ​= ​3). Scale bar: 10 ​mm. F) ELM (LUX_lac_-MCred) response upon PAI sensing from 15 bronchial aspirate samples with non-*P. aeruginosa* infection (12 samples, evaluated without PAI or spiked with 400 ​nM of PAI) and with *P. aeruginosa* infection (3 samples). ELMs were stored at 2–8 ​°C for less than 15 days before use. The a∗ values quantifying red pigmentation are reported as individual data points for each infection group (Gram positive, non-*P. aeruginosa* Gram negative, no detectable CFUs, *P. aeruginosa*; N ​= ​1) and the bars indicate their range. The pairwise comparison between values of spiked vs non-spiked condition in individual samples is reported in Fig. S6. The values reported in panel F and Fig. S6 were measured under a different illumination setup from the other ones reported in this study.

**Fig. 3:**
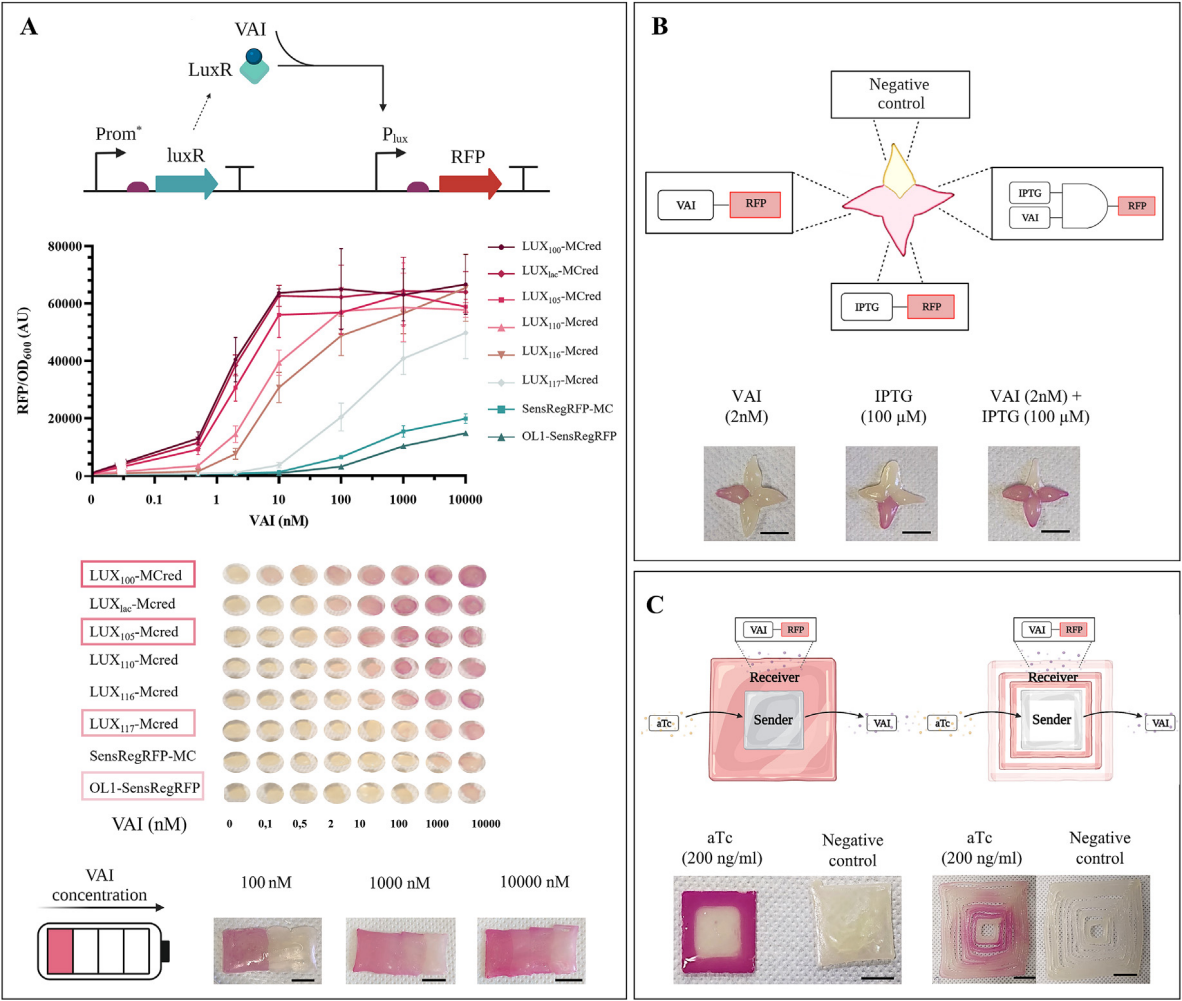
Multi-strain ELMs. A) Level-bar detection of VAI. The panel shows the full prototyping process of this multi-strain material: first, a library of VAI-sensing strains was constructed and characterized in liquid cultures. The graph reports the per-cell fluorescence as a function of VAI concentration in liquid culture, with data points representing average values of three independent measurements and error bars representing standard deviations. Second, the same strains were included in manually extruded structures which were incubated overnight on LB agar containing the indicated VAI concentrations. The picture shows the pigmentation as a function of strain and VAI concentration and the four strains selected for the designed material are highlighted. Finally, a level-bar structure was printed, using the four strains selected above. In the scheme of circuit variants shown on top, Prom∗ are constitutive promoters of diverse strengths, indicated in Table 1; curved arrows: promoters, straight arrows: genes, half-ovals: RBSs, T-shapes: transcriptional terminators, light blue diamond: LuxR protein, blue circle: VAI. B) Independent detection and signal processing in a four-strain ELM with a shuriken shape. The four strains are: a VAI biosensor (LUX_lac_-MCred, left point), an IPTG biosensor (LAC-MCred, bottom point), an AND gate with VAI and IPTG as inputs (SensRegRFP-MC, right point), and a negative control without fluorescent reporters (top point). The pictures show the ELM at day 1 after overnight incubation on LB agar supplemented with the indicated inducers. C) Patterned gene expression in a two-strain ELM using sender (TET-MCvai) and receiver (LUX_lac_-MCred) strains. Pictures are shown for two designs in which sender and receiver are printed in adjacent or slightly separated compartments. Patterned and control structures refer to the same designs with 200 ​ng/ml of aTc or without inducer. In all panels, pictures are shown along with the qualitatively expected output. Scale bar: 10 ​mm.

The LUX_lac_-MCred strain, able to detect the VAI signaling molecule and having RFP as output, was used as a model system to evaluate the performance of a sensing ELM. First, the dose-response curve of the engineered strain was characterized by fluorimetric assays in liquid cultures that showed a per-cell fluorescence increase at VAI concentrations as low as 0.5 ​nM (Fig. 2A). The bioink including this sensor was then prototyped with manually extruded materials that were exposed to different concentrations of VAI on agar plates, and produced gradually increasing amounts of RFP, visible as red pigmentation (Figs. 2A and 3A). Comparison between liquid and solid cultures allowed to measure the RFP expression range that corresponds to an RFP pigmentation visible at naked eye and quantifiable via image analysis (Fig. S4). The lower visible limit of detection (LOD) was between 0.5 and 2 ​nM of VAI and occurred for per-cell fluorescence values of about 15,000 AU in liquid media. This relation persists for overnight incubated ELMs, while longer periods (>36 ​h) correspond to lower LODs due to RFP accumulation (data not shown). Tunability of the dose-response curve is possible by tuning the expression of the transcriptional activator LuxR, or by replacing the reporter gene with one producing more intense color. In this work, both interventions have been carried out and described in sections 3.2.4 and 3.3. Addition of a fast-degradation tag to reporter protein is also possible to increase its turnover and prevent protein accumulation; compared with non-tagged proteins, this intervention would enable the on-to-off transition of biosensors for real-time sensing tasks, but inevitably resulting in a lower per-cell fluorescence and, accordingly, the LOD may increase.

Based on the data above, full induction conditions were applied to the bioprinted sensing patches by using 400 ​nM of VAI included in LB agar. This ELM was able to produce a clearly visible red color after overnight incubation upon induction at day 0, during which bacteria grow and RFP expression is triggered (Fig. 2B). The biosensing ELM was then tested for continuous monitoring of VAI presence: based on our workflow for cell viability maintenance, the ELMs were subcultured by moving the structures on fresh LB agar every four days, and induction was tested up to day 8 on previously unexposed ELMs (Fig. 2B). Regardless of the induction day, an intense pigmentation always occurred and unexposed ELMs did not show a significant leakage of RFP, demonstrating that the VAI sensor is fully functional over a long operation time. The red color took about 3 ​h from induction to develop (Fig. 2B). This time is probably due to different kinetic steps, such as VAI diffusion through the material and cells, promoter activation, gene expression dynamics (transcription and translation), and RFP maturation, the last being an important rate-limiting step with a time constant of about 1 ​h [43]. The use of other fast-maturation reporter proteins exhibiting intense pigmentation is expected to decrease both response time and limit of detection (LOD).

To test storage conditions relevant to field operations, VAI-sensing ELMs printed at day 0 were stored in a refrigerator at 2–8 ​°C for 1 month. VAI sensing was still fully functional upon use, after moving the ELM on LB agar with VAI (Fig. 2B).

In addition to full induction conditions, a dose-response curve was also characterized for VAI-sensing bioprinted patches, by quantifying red pigmentation at different VAI levels (Fig. S5A). Data showed a qualitatively consistent curve compared with manually extruded materials (Fig. S4) also confirming the LOD value of 0.5 ​nM, for which ELM output was statistically higher than the no-induction condition (p ​< ​0.05, *t*-test). The batch-to-batch and day-to-day variabilities of red pigmentation output in patches from independent bacterial batches cultured in the same day and in different days, respectively, were quantified. Experiments showed a similar and modest variability among different bacterial culture batches (CV ​= ​14%) and among different days (CV ​= ​17%), highlighting the reliability of the biosensing devices (Fig. S5A). As expected, the observed red color intensity strongly correlated with red fluorescence (r ​= ​0.97, p ​< ​0.05), quantified via plate reader, showing consistent outputs with different detection methods by biosensor users (Fig. S5B).

#### 3.2.2 Biosensing under non-optimal incubation conditions

The biosensing experiments described above relied on chemical inducers added to solid medium at the desired concentrations by plating. However, practical applications of samples on ELMs may benefit further streamlining, without adopting large amounts of solid media and without the need of plating the samples to be analyzed. To this aim, we carried out biosensing assays 1) in absence of solid media using patches previously grown on LB agar and then removed, and 2) with a small amount of solid media cut from LB agar, by pipetting a small drop of inducer-containing sample, and incubating the ELM at room temperature or at 37 ​°C. All the four conditions resulted in detectable red pigmentation at the maximum VAI concentration tested of 1000 ​nM (Fig. S3A), significantly higher than the non-induced ELMs (p ​< ​0.05, *t*-test), demonstrating that biosensing patches can work in non-optimal conditions with 2.5- to 40-fold activation range. Incubation at room temperature systematically gave less pigmentation than incubation at 37 ​°C (p ​< ​0.05, ANOVA), and incubation of patches on LB agar media gave significantly higher pigment intensity than in absence of solid media (p ​< ​0.05, ANOVA). A significant interaction between temperature and media availability also persisted (p ​< ​0.05, ANOVA), with the presence of LB medium at 37 ​°C providing superior color development compared with the other conditions. Although bioprinted ELMs showed responsiveness and highly reproducible output in all the tested conditions, the resulting dose-response curves were different (Figs. S3A–B). Curves in Fig. S3B were also different from the response of the same sensor tested on LB agar plates (Fig. S5), probably due to the different final concentration of VAI between agar plate and applied sample drop. For these reasons, a dramatic variation of biosensing capabilities in terms of LOD value is expected among largely different experimental conditions. For comparison, an output intensity similar to that of agar plate assays at the LOD concentration (i.e., 0.5 ​nM of VAI, see Fig. S5) was reached at a VAI concentration of 10 ​nM for the ELMs in the cut agar condition at both 37 ​°C and 25 ​°C (Fig. S3B). In the 37 ​°C with no agar condition, a similar value was reached at 1000 ​nM of VAI, and for ELMs at 25 ​°C with no agar it was never reached in the tested VAI range (>2-fold difference, Fig. S3B).

This feature motivates the need of defining and testing a range of working conditions for ELMs, in which their capability to sensitively discriminate between the target conditions is still acceptable. In qualitative on/off sensing applications, a maximum LOD value should be quantified and molecule concentrations above this threshold can be reliably detected thanks to the high reproducibility of output pigmentation, herein demonstrated (Fig. S3 and Fig. S5). More quantitative detection tasks relying on calibration curves could also be affected by environmental conditions, requiring proper measurements from ELMs incubated in the same condition as in the target assay.

To gain further insight into condition-dependent output expression, cell viability was measured for all the conditions above at the end of the experiment. Data showed a strong correlation (r ​= ​0.98, p ​< ​0.05) between red pigmentation intensity and final cell density in the ELM (Fig. S3C), meaning that the variation in RFP expression can be explained by cell growth, but the regression line has a non-null intercept and for this reason no proportionality persists between the two measured variables.

The feasibility of incubating ELMs at room temperature without specific nutrients will pave the way to biosensing tasks by ELMs in environmental niches, while application of samples to the ELM in the 37 ​°C ​+ ​LB condition will be used as an easy-to-use kit to analyze clinical samples. These two situations were tested in sections 3.2.3 and 3.2.4, aiming to evaluate if ELM induction can occur in field relevant matrices.

#### 3.2.3 Biosensing inside environmentally relevant samples

VAI-dependent induction was tested by placing ELMs on (or within) VAI-containing soil and immersing them in VAI-containing tap water at room temperature. The sensors showed induction when placed both on the surface and when immersed in soil (Fig. 2C). Although the induced materials showed clearly distinguishable color compared with negative controls without VAI, the color was less intense than the one developing on VAI-containing LB agar, probably due to the lower amount of nutrients in soil than in LB and to the resulting lower concentration of VAI (see section 3.2.2). Induction in tap water was also functional (Fig. 2C). However, red color could not be observed immediately after removing them from the water, and additional 2 ​h were needed after removal to let the red color develop at room temperature, probably due to RFP maturation which requires oxygen to occur. After that time, the visible color was weak (data not shown) and an intense one could be appreciated only after a further incubation at room temperature. Again, a change of reporter protein could overcome the oxygen requirement and provide a clearly detectable output in a shorter time. These data demonstrate that on/off sensing could effectively occur in field-relevant conditions, in which temperature and nutrients are not optimal for bacterial growth and gene expression. Soil is prone to contaminations by competing microbes due to its composition and immersion in water can result in nutrient washout from ELMs. Also, incubation in a refrigerator for a long time may cause dehydration or crosslinking failure. These challenging conditions were a successful testbed for the fabricated living materials.

#### 3.2.4 Biosensing in clinically relevant samples

The VAI-sensing ELM was used to detect a signaling molecule produced by pathogenic bacteria. In fact, this sensor is also capable of detecting PAI from *P. aeruginosa*, exploiting non-specific activation of the lux-based machinery by this molecule (Fig. 2A and Fig. S5A). Based on per-cell RFP values in liquid cultures and red pigmentation of ELMs on agar plates, the LOD of PAI increases significantly compared with VAI, resulting in a limit of ∼10 ​nM (Fig. 2A and Fig. S5A), for which ELM output becomes statistically higher than the no-induction condition (p ​< ​0.05, *t*-test). The sterile-filtered supernatants of 4 clinical isolates of *P. aeruginosa* grown to saturation were collected and applied to biosensing patches, previously stored in a refrigerator for 1 day, with a cut agar section. After incubation at 37 ​°C, red pigmentation was clearly visible for 2 samples and weakly visible for the others (Fig. 2E), all statistically higher than the negative control output (p ​< ​0.05, *t*-test), demonstrating the capability of such ELM to detect molecules directly produced by pathogenic bacteria. By using liquid biosensing assays, the output diversity in the samples was confirmed to be due to differences in PAI production levels among the clinical isolate cultures, with concentrations of ∼1 ​μM for the isolates giving the highest RFP output, and under the detection limit of the assay for the others.

As a second test on clinically relevant samples, we used minimally processed bronchial aspirate samples from patients affected by non-*P. aeruginosa* (N ​= ​12) and *P. aeruginosa* (N ​= ​3) infections. Processing included addition of a solution to decrease viscosity, routinely added upon sampling, and a centrifugation step to retrieve the (unfiltered) supernatant fraction. The non-*P. aeruginosa* samples were also spiked with PAI at 400 ​nM to confirm the feasibility of PAI detection in complex matrices. Samples were applied to ELMs and incubated as before. None of the non-*P. aeruginosa* bronchial aspirate samples triggered a relevant red pigmentation, demonstrating high biosensor specificity for other Gram negative and Gram positive pathogens (Fig. 2F and Fig. S6). A large part of the spiked non-*P. aeruginosa* bronchial aspirate samples (10/12) showed red pigmentation, demonstrating successful detection of PAI in highly complex samples in which growth-inhibiting compounds may be present (Fig. 2F and Fig. S6). Finally, one of the three samples with *P. aeruginosa* also gave visible red pigmentation (Fig. 2F and Fig. S6). While no information was available in terms of bacterial density or PAI concentration in bronchial aspirates, it is not surprising that a large variation occurs among clinical samples, in which a wide range of PAI levels was reported, from picomolar to low micromolar concentrations [44]. Other synthetic circuits were proposed to detect PAI with lower LOD values [31,45] and the bioprinting techniques used in this study may serve in conjunction with such circuits to facilitate the construction, delivery and approval of sensing devices for clinical use, starting from matrices of different complexity and with minimal sample processing efforts.

Overall, the construction of living devices with functioning compatible with real-life applications was demonstrated: the data on single-sensor ELMs demonstrate that effective sensing materials could be engineered, with attractive features beyond the laboratory setting, including successful functioning over time, in harsh conditions, in complex matrices and detecting a compound relevant for healthcare. Our workflow also enabled the fabrication of other ELMs with different sense-and-respond functions: IPTG-inducible synthesis of a biopolymer (γ-PGA) with an engineered strain of *B. subtilis* (Fig. S7), showing compatibility with a different microbe, and tetracycline sensing with engineered *E. coli* expressing a weakly-visible and poorly-reproducible red pigmentation (TET-MCred strain, Fig. 2A) or a strong yellow pigmentation (TET-MCyellow, using the catechol 2,3-dioxygenase xylE gene instead of RFP - Fig. 2D), showing genetically-designed improvement of ELM reporting capability at naked eye.

The illustrated results highlighted that the developed devices can meet key biosensor criteria such as easy assembly and readout acquisition, evolutionary stability and reproducibility. Regarding their performance, sensitivity was assessed in terms of LOD during ELM characterization on inducer-containing agar plates. However, this parameter is influenced by several environmental factors investigated in this work such as temperature, nutrient and oxygen availability, and sample matrix that may include inhibitors; to counteract performance decreases, sensitivity could be improved by users via genetic design (e.g., tuning transcriptional regulator expression level and reporter gene choice), readout measurement technology (e.g., image or fluorescence acquisition system) and detection assay conditions (e.g., tuning the pipetted volumes). Characterization of biosensor ELMs in conditions similar to the working context is expected to elucidate the application-dependent sensitivity of the fabricated devices, to possibly reach reasonable detection limits.

On the other hand, specificity is expected to be mainly dependent from the unwanted crosstalk affecting the genetic components (e.g., transcriptional regulator, promoter and reporter gene) and from the sample matrix composition. In this work, no false positive was observed in ELM outputs when exposed to no-inducer conditions. Nonetheless, unspecific activation of a VAI-sensitive device by PAI, produced by *P. aeruginosa*, was exploited to define a pathogen detection kit in real samples, but crosstalk by other bacterial autoinducers may affect its specificity. Moreover, in the detection assay with clinical samples we observed a few non-*P. aeruginosa* bronchial aspirates giving high background, probably due to intrinsic pigmentation caused by hematic content in some of the samples. In our study, such background was always lower than the output of spiked samples showing RFP production, but this effect could be relevant to estimate the false positive occurrence on larger number of samples.

### 3.3 Multi-strain biosensing materials

We fabricated materials composed of multiple strains, addressing three key applications of engineered living materials: quantitative or semi-quantitative output reporting, multiplexed sensing, and interacting microbial strains driving pattern formation.

Quantitative output reporting is of particular interest to measure compound concentration in field contexts in which the input level saturates the sensor output, or in which a trivial quantification of the produced color can be highly variable and poorly informative to understand the actual concentration sensed. To mitigate this issue, we designed a level-bar ELM composed of four different strains with diverse LODs for the same molecule (VAI). To this aim, we constructed a library of isogenic VAI-sensing strains exhibiting different dose-response curves by placing the LuxR-coding gene under the control of constitutive promoters with graded strengths from the Anderson collection (Table 1). An initial library of 8 strains was screened in liquid culture assays and in manually extruded constructs on solid media at the specified VAI inducer concentrations to assess their difference in LOD (Fig. 3A and Fig. S4). Then, four of these strains, showing significant diversity in LODs, were selected for printing a bar material able to trigger red pigmentation in some of the strains depending on VAI concentration (Fig. 3A). Alternative designs could be also adopted without bioprinting multi-strain materials, with the use of an individual strain at different concentrations of the sample containing the compound to be sensed, or the use of physically separated wells for each strain, also applicable for multiplexed sensing [32]. However, the realized multi-strain solution does not require substrate manipulation, is compatible with rapid prototyping and automated construction, and could be able to provide a discrete quantification of the sensed molecule against a standard curve constructed in the same matrix. When calibration curves cannot be constructed, level-bar ELMs could be adopted as semi-quantitative tools indicating discretized low-to-high output categories to be compared with the output bar of other samples, enabling comparisons but not quantification of the target molecule.

Multiplexed sensing was demonstrated by constructing materials following a shuriken-like structure including strains with independent RFP-producing sensors and information processing circuits. The resulting ELM could successfully sense two inputs (VAI and IPTG) and provide colorimetric outputs resulting from their processing (Fig. 3A). The four compartments of the ELM act as 1) negative control (non-fluorescent strain), 2) VAI sensor (LUX_lac_-MCred), 3) IPTG sensor (LAC-MCred), and 4) an AND gate with VAI and IPTG as inputs (SensRegRFP-MC), respectively. This demonstrates that complex patterns could be effectively realized by spatial control of individual strains to engineer materials with multiple outputs.

Finally, we tested ELMs in which multiple strains interact with each other. Even though bioprinting platforms enable the manufacturing of complex patterns in the materials, an additional level of complexity can be enabled by interactions between communicating bacterial strains [2,46]. To demonstrate this possibility, we printed a material composed of two bioinks. One of them includes a *sender* strain (TET-MCvai), able to synthesize VAI upon induction with Tc or aTc. The other one includes one of the VAI-sensing library members (LUX_lac_-MCred) described above that acts as a *receiver* strain. The receiver was expected to form a visible RFP pattern due to the diffusion of VAI through the compartments and the growth medium. Patterns of gene expression were successfully visualized via red pigmentation in the receiver compartment upon aTc induction of the sender strain, in structures in which VAI diffusion occurred between strains in adjacent compartments of the structures or occurred mainly through the solid medium on which the two-strain ELM is placed (Fig. 3C). The output expression strength could be tuned by changing sender/receiver material geometry, sender induction level and LOD of the receiver strain, thus providing a toolkit for expression pattern formation.

Taken together, these data demonstrate the benefits of rapid prototyping complex multi-strain materials for different applications that were herein used to address key aspects in the biosensing field.

## 4 Conclusions

A bacterial bioprinting workflow was adopted to investigate two key outstanding aspects required to streamline the use of engineered living biosensor materials for real world applications: compatibility with field use in different scenarios, and engineering function complexity by materials embedding multiple bacterial strains.

Efficient biosensing of chemicals succeeded in nutrient-poor conditions such as soil or water, relevant for the monitoring of molecules in environmental niches, and in complex matrices such as culture supernatants and bronchial aspirates from patients, relevant for the development of rapid detection kits in clinical samples, with the aim of demonstrating the feasibility of on/off induction in such complex sensing conditions. Field-relevant features also included the maintenance of cell viability and synthetic circuit stability over two weeks of continuous functioning and the possibility to store the bioprinted materials in refrigerated conditions for more than a month before use. However, dose-response curves of sensors were affected by the environmental context used in detection assays, influencing their sensitivity, and highlighting the need of characterizing their response in conditions similar to the target ones to estimate the application-specific detection limit.

Even though the bioprinting procedure herein used is different from previous reports on bacteria-laden materials, in terms of abiotic component composition, living component content, and printing-crosslinking process, we obtained comparable performances for long-term viability, storage and sensing in water samples, and successfully tested a procedure to prolong cell viability. Our data also showed a remarkable performance of biosensing materials in complex environments such as soil and clinical samples, suggesting that living materials may surpass other technologies such as cell-free approaches, in terms of sensing in complex matrices with no need of sample processing, as we demonstrated with the detection of the *P. aeruginosa* autoinducer in spiked and real clinical samples. Biocontainment, not addressed in this work and already demonstrated in previous works on engineered living materials, will provide deployable devices for field applications with no genetically modified microorganisms escape.

Then, a number of benefits have been demonstrated by fabricating living materials with more than one bacterial strain, prototyping devices that support the (semi-)quantitative detection of a molecule, the multiplexed detection of different molecules or their combination, and cell-cell communication-mediated patterning of materials. The complexity of such functions, enabled by the precise spatial control of strain printing and by synthetic biology tools to engineer new functions, will boost the potential of biosensing and signal processing capabilities in such devices. Synthetic biology interventions have also been demonstrated to properly tune the response of one of the sensors that provided a too low output range and needed an amplification of the visible readout.

Taken together, this work has demonstrated that bacterial bioprinting can be used to fabricate multi-input/multi-output and highly reproducible biosensing devices in terms of shape, performance, longevity, and stability. Such procedure, boosted by the fabrication of material with multiple strains, is amenable to further automation to streamline the whole design and prototyping process of bacteria-laden engineered biosensing materials.

## Credit author statement

FU: conceptualization, methodology, validation, formal analysis, investigation, data curation, writing - original draft, visualization. GL: methodology, investigation, writing - review & editing, visualization. FS: methodology, formal analysis, investigation, data curation, writing - review & editing. MB: validation, investigation. IC: resources. MC: conceptualization, methodology, resources, writing - review & editing, supervision. LP: conceptualization, methodology, formal analysis, investigation, writing - original draft, supervision. All authors have read and agreed to the published version of the manuscript.

## Declaration of competing interest

The authors declare that they have no known competing financial interests or personal relationships that could have appeared to influence the work reported in this paper.

## Data Availability

Data will be made available on request.
