# Supplementary material for: Design and biofabrication of bacterial living materials with robust and multiplexed biosensing capabilities
Source: Mater Today Bio. 2022 Dec 24;18:100526. doi: 10.1016/j.mtbio.2022.100526 (PMC9826803; doi:10.1016/j.mtbio.2022.100526)
Supplement: Multimedia component 1 [file mmc1.pdf]

Supporting information

Design and biofabrication of bacterial living materials with robust and multiplexed biosensing capabilities

Text S1 - Details on the bioprinting workflow definition process

The final procedure, described in the Methods section of the main text, includes a hydrogel with sodium alginate and gelatin at the 8% (w/v) and 4% (w/v) concentrations, respectively [40], due to its printability and handling of the resulting structures. The crosslinking step was carried out by adding calcium chloride to the bioink extruded on a flat plastic support (e.g., a Petri dish). This crosslinking procedure has been used previously but not for bacteria. Compared with previous studies on bacterial ELMs adopting similar abiotic components [15; 16; 20], we used a higher hydrogel concentration to allow for easy handling (e.g., moving onto different solid or liquid media) of even thin bioprinted structures with less than 1 mm height, which could be robustly taken with a spatula or a scalpel. Another convenient technique enabling direct crosslinking upon deposition of the extruded bioink onto solid medium supplemented with calcium chloride was tested [15], but resulted in frequent nozzle clogging due to premature gelation upon contact between nozzle and non-flat agar plates surface.

Preliminary tests aimed to select bioink composition in terms of hydrogel and nutrients, growth conditions for ELMs, printing support, and crosslinking procedure (Figure S1). Among the variables tested, hydrogel composition and concentration did not affect cell growth; the nutrient content in the bioink slightly affected it without any visible change in RFP production. The main factor affecting RFP production was the presence of external medium nutrients, which were required to achieve intense red pigmentation. This requirement was expected since bacterial density was slightly lower than previous studies and nutrients could allow for bacterial proliferation after printing [15; S1].

Text S2 - Quantification of printing fidelity and reproducibility

The shape fidelity and reproducibility of printed structures was quantified by analyzing grids at different heights and distance between filaments (Table S1). Low variability in filament width and distance was observed, with a CV always lower than 30% among replicated measurements in each analyzed geometry (Figure S2). The average filament width values were about 1.2 mm; they showed a low variation among structures with different heights (CV=17%) and distance between filaments (CV=12%), with a slight increase of width (1.6 mm) in the 1.8-mm tall structures (Figure S2A,D), as previously reported with other platforms [15]. In our procedure, this increase is most probably due to bioink spreading upon layer addition before crosslinking. The average distance between filaments

varied predictably with the computational design (Figure S2B), accounting for the average filament width measured above, and showed low variation (CV=5%) for different structure heights (Figure S2E). In the latter case, the average distance values (about 3 mm) were not affected by the slight increase of filament width in the highest structures, described above (1.2 to 1.6 mm), probably because this observed difference (about 0.4 mm) was comparable with the standard deviation of the distance values (0.37 mm). The bioprinting performances were not altered in absence of bacteria in the bioink (Figure S2G), as expected from previous studies with different abiotic and living components [S2].

The hollow parts of the fabricated grids had the expected square shape in the lowest-height highest-distance grid (Pr=0.99). In all the other cases, the Pr value remained around 0.9, demonstrating a reasonable predictability in pore shape (Figure S2C,F).

Finally, the weight of structures at day 1, measured on a batch of bioprinted grids, showed an extremely low variability (CV<19%), indicating that the inter-ELM volume of extruded bioink is highly repeatable (Figure S2H).

Supplementary references

[S1] Schmieden DT, Basalo Vazquez SJ, Sanguesa H, van der Does M, Idema T, Meyer AS. Printing of patterned, engineered E. coli biofilms with a low-cost 3D printer. *ACS Synth Biol.* 7:1328-37, 2018. doi: 10.1021/acssynbio.7b00424.

[S2] Balasubramanian S, Yu K, Meyer AS, Karana E, Aubin-Tam ME. Bioprinting of regenerative photosynthetic living materials. *Adv Funct Mater.* 31:2011162, 2021. doi: 10.1002/adfm.20201116.

Supplementary figures and tables

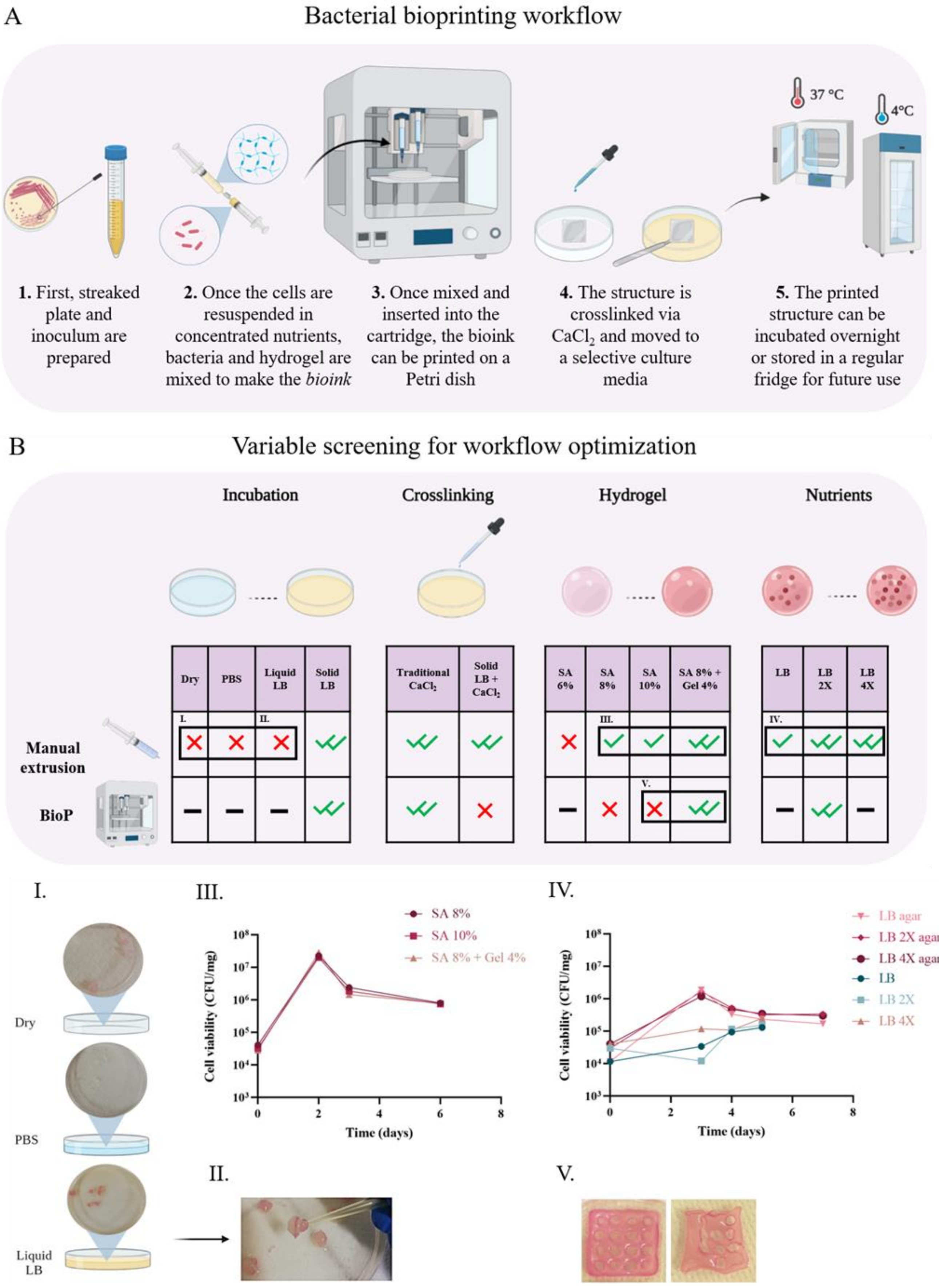

Figure S1. Bacterial bioprinting workflow used in this work and screened variables. A) Scheme illustrating all the steps of the defined workflow, described in the Methods section of the main text, based on the screening results below. B) Main variables screened during protocol development: incubation, crosslinking, hydrogel composition, and nutrient concentration. Screening outcomes are reported as double ticks, single ticks, and crosses, qualitatively corresponding to a good condition, a reasonably good condition but not the best among the tested ones, and a non-satisfying condition, respectively. These results are relative to screening procedures carried out with manually extruded or bioprinted structures, as indicated. For bioprinted structures, the minus sign corresponds to non-tested conditions. The reported pictures and graphs in subpanels show examples supporting the selection of the variables, as described below. All the screening experiments were carried out in single or duplicate tests, using HCred as the living component.

Among the hydrogel conditions tested, the 6% (w/v) alginate concentration gave qualitatively poor structural tone in manually extruded and crosslinked inclusions compared with the other hydrogels, not allowing reliable handling (data not shown), and was not further considered for bioprinting. The other hydrogels gave reasonably satisfactory results, with the addition of gelatin giving superior toughness.

The manually extruded and crosslinked structures with two different hydrogels (8% (w/v) sodium alginate and 8% (w/v) sodium alginate + 4% (w/v) gelatin, both containing 2-fold concentrated LB nutrients) were incubated on solid LB, liquid LB medium, plastic, or PBS. The two conditions with the exogenous LB supported intense color development, suggesting that additional energy sources to the medium components in the bioink were necessary to support efficient growth and/or expression (panel B-I). Solid medium was further selected, consistent with previous works, since incubation in liquid media caused a rapid contamination of the liquid by planktonic growth, probably depleting some nutrients, and resulting in a more variable color development across the experiments and in different parts of the inclusions (panel B-II). In addition, all the hydrogels supported cell growth and protein expression in the tested conditions (2-fold concentrated LB in the bioink, incubation on solid LB), based on colony count and visual inspection of intense red color: no difference in cell viability profile was observed over about a week on solid medium in alginate concentrations at 8% and 10% (w/v), and at 8% (w/v) with the addition of 4% (w/v) gelatin (panel B-III). Removal or addition of LB nutrients had a low impact on cell viability (panel B-IV), evaluated with a 8% (w/v) sodium alginate + 4% (w/v) gelatin bioink, confirming that the exogenously provided nutrients play a major role; among the LB nutrient levels tested in the bioink, the 2-fold concentrated LB condition was selected since it supported a slightly higher cell viability than the standard LB (panel B-IV). The data in panel B-IV further support the selection of solid medium instead of liquid medium due to the superior cell viability over time per mg of dissolved material.

Crosslinking was successfully carried out with manually extruded structures by applying a drop of calcium chloride for 5 min (see protocol in main text), or by directly extruding the bioink on calcium chloride-supplemented LB. However, the latter procedure failed in the bioprinting because of frequent nozzle clogging probably due to non-perfectly flat media surfaces (see main text) and it was not further adopted. Finally, bioprinting tests confirmed that the gelatin-including hydrogel could be handled more reliably after crosslinking, without disrupting the structure (panel B-V).

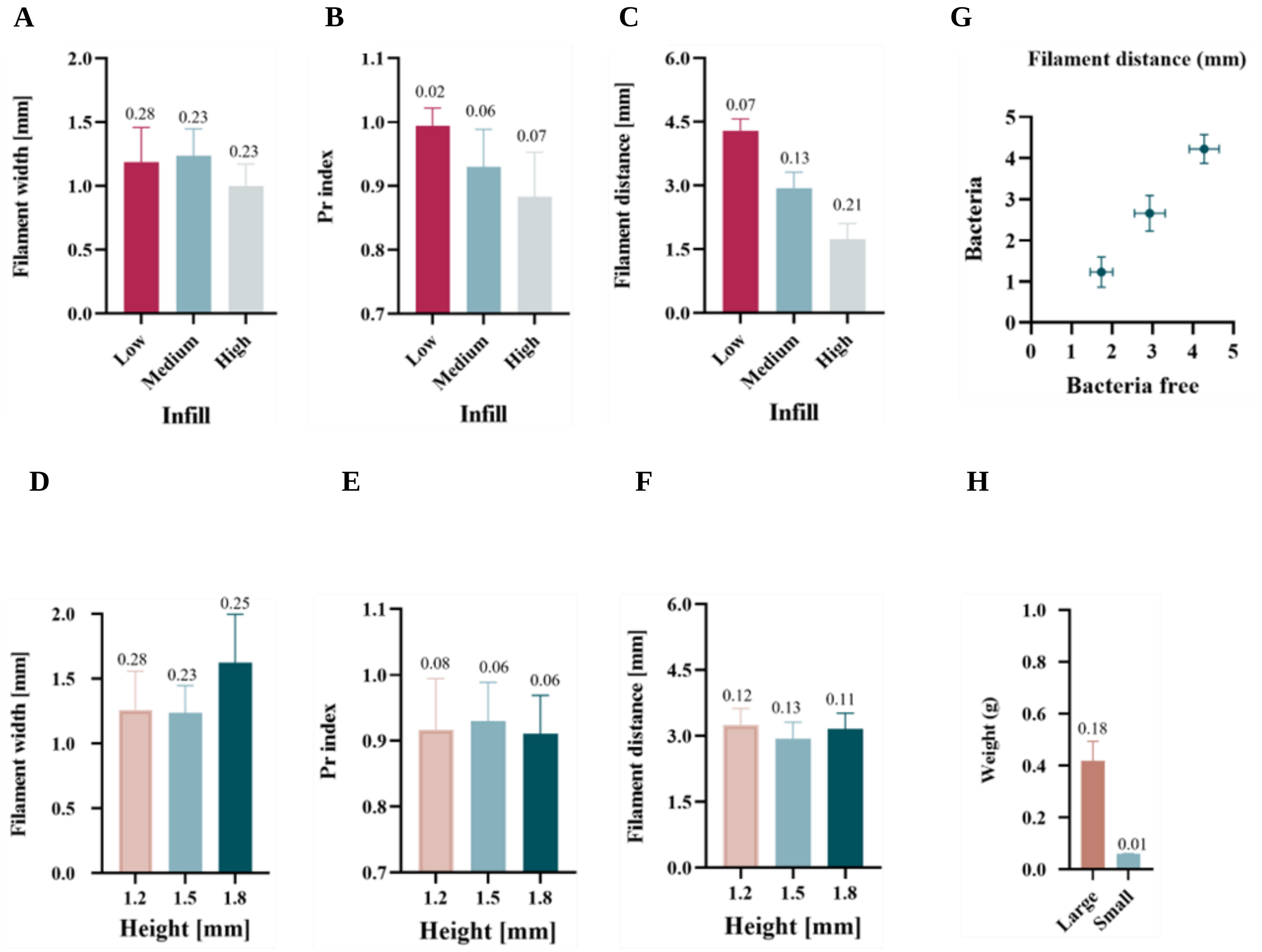

Figure S2. Measured printability indexes. Bar charts showing A) filament width, B) Pr index, and C) distance between filaments for grid structures with 1.5-mm height, printed at different infill density (low, medium, high; see Table S1). Bar charts showing D) filament width, E) Pr index, and F) distance between filaments for grid structures printed at medium infill density at different heights (1.2, 1.5, 1.8 mm; see Table S1). G) Scatter plot showing the relation between filament distance measurements in structures with and without bacteria. H) Bar chart showing the weight of 20x20x2.5 mm grids including the PB5741 strain (Large; N=17) and 10x10x0.7 mm grids including the MCred strain (Small; N=3). In all panels, bars and data points represent the average values and error bars represent standard deviations. In panels A-G, the coefficient of variation (CV) is reported for each bar (N>30).

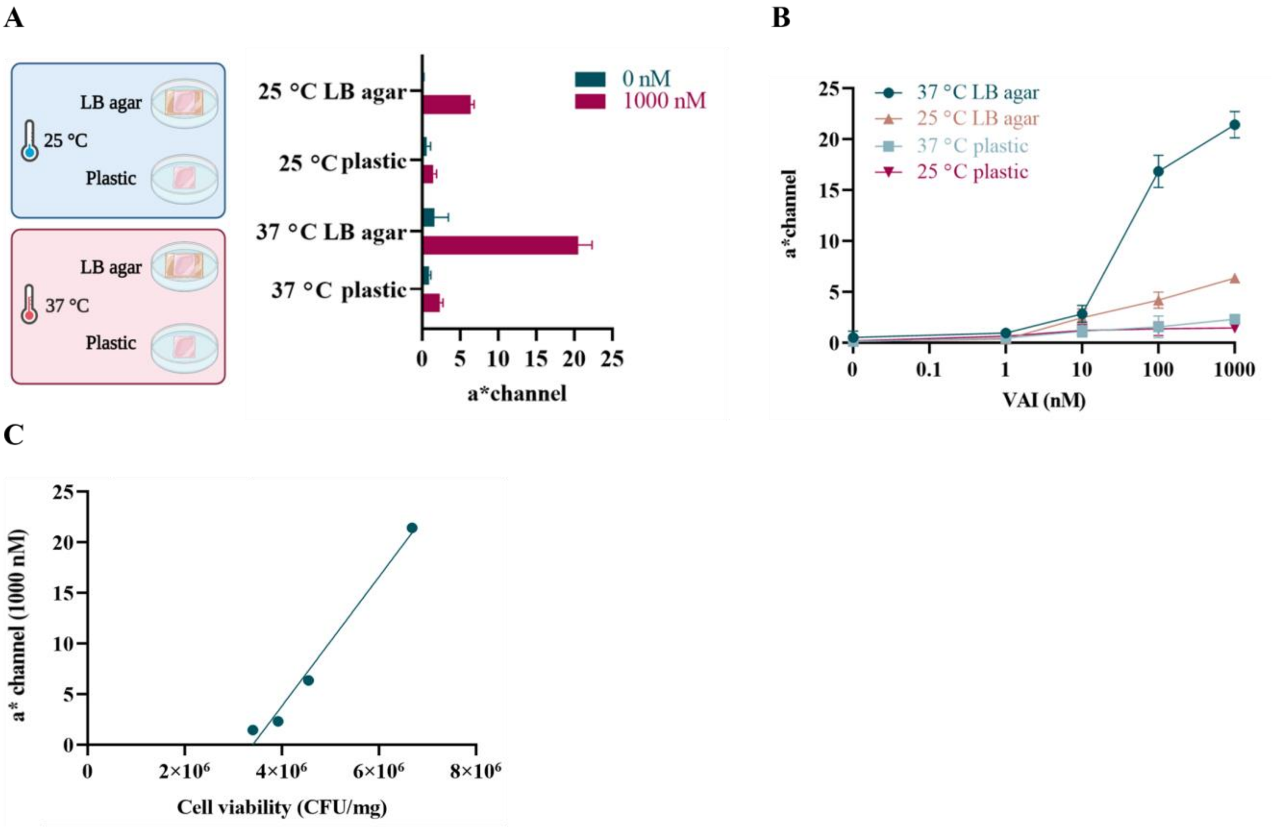

Figure S3. VAI-sensing ELM response upon incubation on plastic or cut agar with a 200-µl drop of autoinducer at the indicated concentrations. The four experimental conditions are summarized on the left, namely two temperatures (room temperature - about 25 °C, vs. 37 °C) and two nutrient conditions (ELM on plastic Petri dish with no additional nutrients, vs. ELM with a small piece of cut solid medium - LB agar). In the condition without additional nutrients (plastic), ELMs printed at day 0 were incubated overnight on solid medium (LB agar), then moved to a Petri dish at day 1 and incubated overnight in presence of VAI samples; ELM response is then evaluated at day 2. In the condition with cut LB agar, ELMs printed at day 0 were incubated on cut LB agar in presence of VAI samples; ELM response is then evaluated at day 1. A) Quantification of the red pigmentation, in terms of a* values, in each condition without VAI and with 1,000 nM of VAI. Bars represent mean values with the standard deviations as error bars (N=3). B) Dose-response curves, in terms of a* values as a function of VAI concentration, of the ELM in the different conditions tested. Data points represent mean values with the relative standard deviations as error bars (N=3). C) Relationship between cell density in the ELMs (CFU/mg) and intensity of red pigmentation (a* values). Circles represent mean values (N=3) for cell viability and red color intensity. Solid line represents the linear regression fitting of the mean values. Replicates refer to different bacterial culture batches prepared in in the same day and used to print ELMs for each VAI concentration tested.

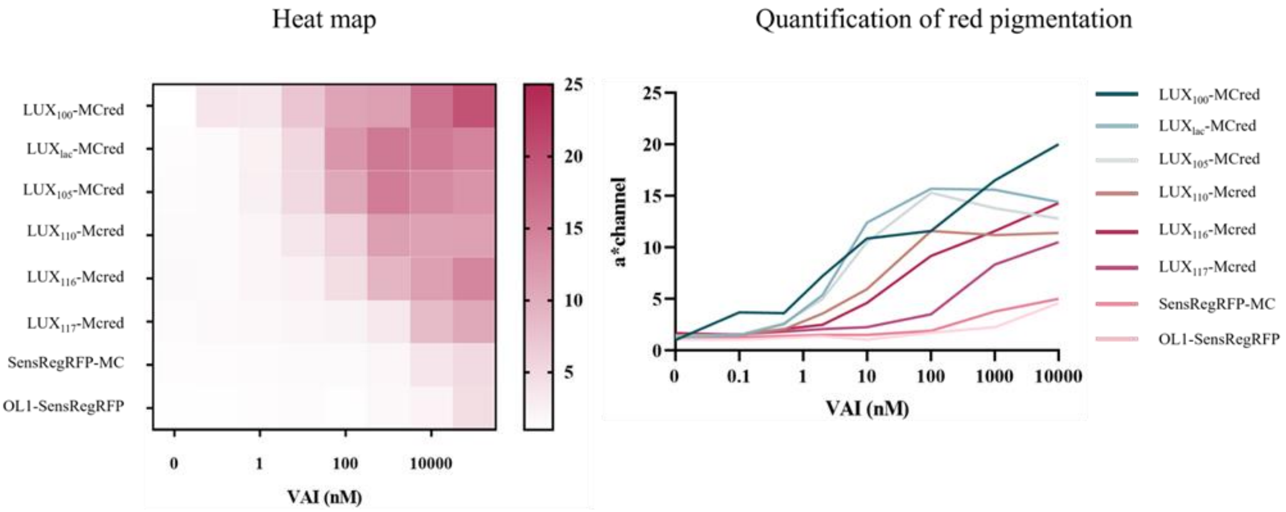

Figure S4. Quantification of red pigmentation (a* values) on inclusions with the indicated biosensor strains, which differ for the expression level of LuxR and result in diverse switch points and LODs. The same a* values are presented as a heatmap and as dose-response curves, at the indicated VAI concentrations. Data come from a single replicate of the strains tested in parallel on the same day. The response of the sensors was evaluated by manually extruding and crosslinking bacteria-laden inclusions at day 0, applying the inclusions to LB agar containing the indicated VAI concentrations, and incubating the structures overnight at 37 °C; response was evaluated at day 1.

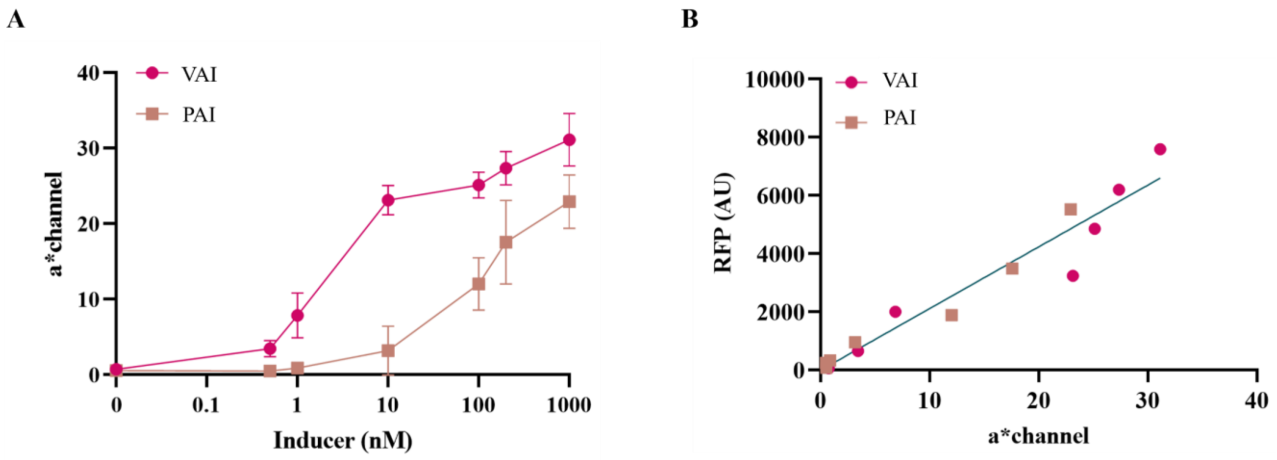

Figure S5. Reproducibility of ELM response. A) Dose-response curves of bioprinted ELMs including LUX_{lac}-MCred for different VAI and PAI concentrations in LB agar plates. ELMs were stored at 2-8 °C for 1 day before use. Circles represent mean values and error bars represent standard deviations (N=8). Replicates refer to different bacterial culture batches prepared in 3 different days (2, 3 and 3 different batches, respectively) and used to print ELMs for each VAI concentration tested. B) Relationship between red pigmentation (a* channel values, quantified by image analysis) and red fluorescence (RFP values, quantified by plate reader). Circles represent average values (N=8) of a* and RFP. The RFP values were obtained by transferring cut pieces of ELMs into 96-well plates and measuring red fluorescence in 3-by-3 square-filled acquisitions per well (RFP gain = 30) and averaging the obtained values. Solid line represents the linear regression fitting of the average values.

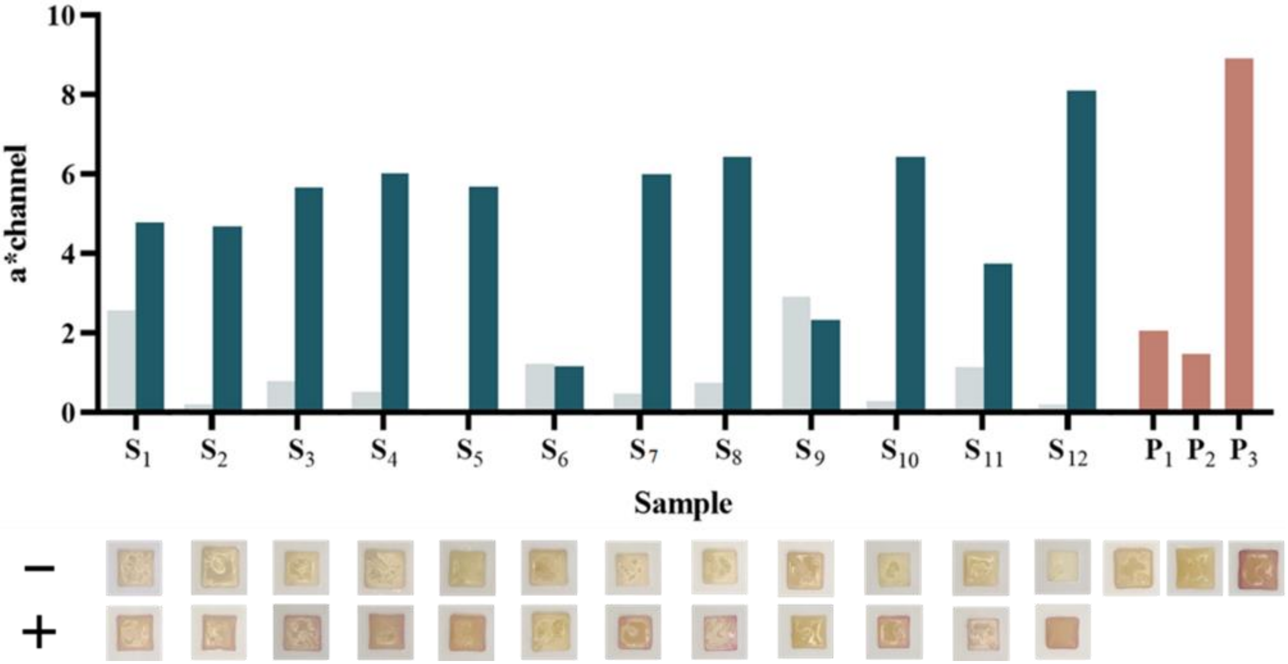

Figure S6. ELM response upon PAI sensing from bronchial aspirate samples from patients. The S1-S3 samples contained non-*P. aeruginosa* Gram negative bacteria; the S4 sample did not yield any detectable CFU; the S5-S12 samples contained Gram positive bacteria; the P1-P3 contained *P. aeruginosa*. ELMs were printed at day 0, moved to LB agar, and stored at 2-8 °C for less than 15 days. Then, LB agar was cut, the ELMs+LB agar pieces were moved to a 6-well plate, and a 200-µl drop of bronchial aspirate sample was applied; ELMs were incubated overnight at 37 °C and then evaluated. The a* values are reported to quantify the red pigmentation and the corresponding pictures are shown below (N=1).

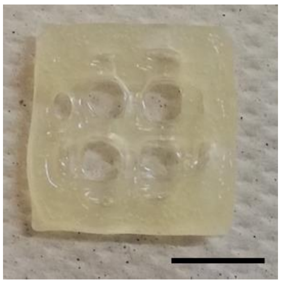

Figure S7. Grid structure including Bacillus subtilis strain PB5741. The PB5741 strain is a derivative of JH642 in which the pgs operon, responsible for poly-γ-glutamic acid (γ-PGA) synthesis, is regulated by the IPTG-inducible Phy promoter. Structures incubated on LB agar containing IPTG showed the typical mucoid phenotype of γ-PGA producers (data not shown). Scale bar: 1 cm.

Table S1.

Geometries and applications of the structures used in this work.

Shape	Dimension	CAD	After overnight incubation	Application
Grid	10x10x0.7 mm	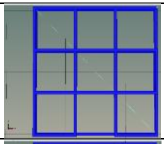	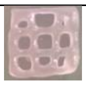	Cell viability test
Grid	20x20x [1.2 up to 1.8] mm	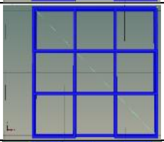	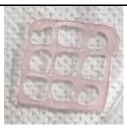	Printability index calculation
Grid	20x20x2.5 mm	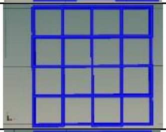	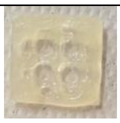	γ-PGA synthesis
Cuboid	20x20x5.1 mm	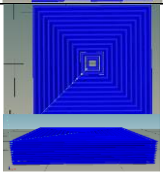	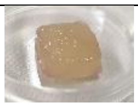	Height test
Thunderbolt	20x30x1.05	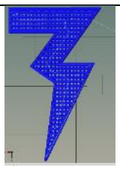	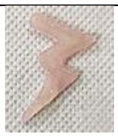	Complex shape
Apple	Side ~ 14 mm	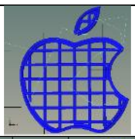	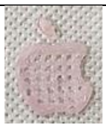	Complex shape
Numbers	Side ~ 11 mm	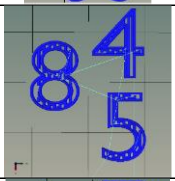	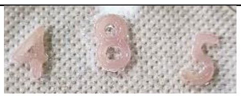	Complex shape
Letters	Side ~ 11 mm	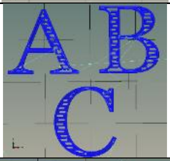	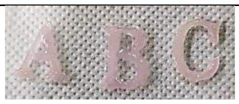	Complex shape
Multi patch	40x20x0.35 mm	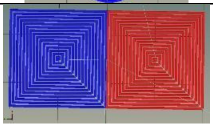	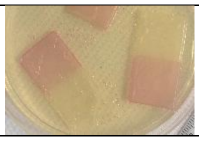	Multi-strain material
Patch	20x20x1.4 mm	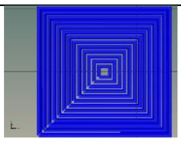	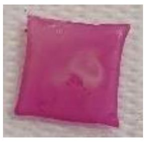	Single-strain biosensor
Letters	Side ~ 11 mm	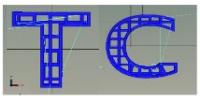	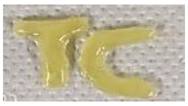	Single-strain biosensor

Level bar	20x40x1.2 mm	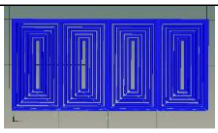	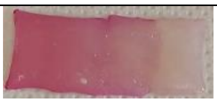	Quantitative multi-strain biosensor
Shuriken	Length single spike ~ 12 mm, height 1.2 mm	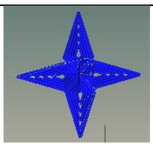	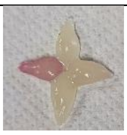	Multi-strain biosensor
Patch with frame	Sender side ~ 12 mm Receiver side ~ 20 mm	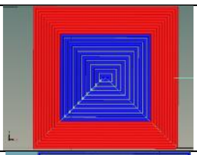	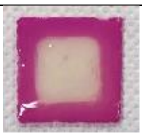	Sender and receiver
Separated frames	Sender side ~ 15 mm Receiver side ~ 50 mm	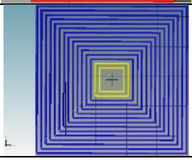	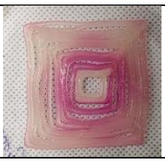	Sender and receiver
